# Whole-genome sequence analysis reveals differences in population management and selection of European low-input pig breeds

**DOI:** 10.1186/1471-2164-15-601

**Published:** 2014-07-16

**Authors:** Juan Manuel Herrero-Medrano, Hendrik-Jan Megens, Martien AM Groenen, Mirte Bosse, Miguel Pérez-Enciso, Richard PMA Crooijmans

**Affiliations:** Animal Breeding and Genomics Centre, Wageningen University, De Elst 1, 6708 WD Wageningen, The Netherlands; Centre for Research in Agricultural Genomics, Consortium CSIC-IRTA-UAB-UB, Edifici CRAG, Campus Universitat Autonoma Barcelona, 08193 Bellaterra, Spain; Institut Català de Recerca i Estudis Avançats (ICREA), 08010 Barcelona, Spain

## Abstract

**Background:**

A major concern in conservation genetics is to maintain the genetic diversity of populations. Genetic variation in livestock species is threatened by the progressive marginalisation of local breeds in benefit of high-output pigs worldwide. We used high-density SNP and re-sequencing data to assess genetic diversity of local pig breeds from Europe. In addition, we re-sequenced pigs from commercial breeds to identify potential candidate mutations responsible for phenotypic divergence among these groups of breeds.

**Results:**

Our results point out some local breeds with low genetic diversity, whose genome shows a high proportion of regions of homozygosis (>50%) and that harbour a large number of potentially damaging mutations. We also observed a high correlation between genetic diversity estimates using high-density SNP data and Next Generation Sequencing data (r = 0.96 at individual level). The study of non-synonymous SNPs that were fixed in commercial breeds and also in any local breed, but with different allele, revealed 99 non-synonymous SNPs affecting 65 genes. Candidate mutations that may underlie differences in the adaptation to the environment were exemplified by the genes AZGP1 and TAS2R40. We also observed that highly productive breeds may have lost advantageous genotypes within genes involve in immune response – e.g. IL12RB2 and STAB1–, probably as a result of strong artificial in the intensive production systems in pig.

**Conclusions:**

The high correlation between genetic diversity computed with the 60K SNP and whole genome re-sequence data indicates that the Porcine 60K SNP Beadchip provides reliable estimates of genomic diversity in European pig populations despite the expected bias. Moreover, this analysis gave insights for strategies to the genetic characterization of local breeds. The comparison between re-sequenced local pigs and re-sequenced commercial pigs made it possible to report candidate mutations to be responsible for phenotypic divergence among those groups of breeds. This study highlights the importance of low input breeds as a valuable genetic reservoir for the pig production industry. However, the high levels of ROHs, inbreeding and potentially damaging mutations emphasize the importance of the genetic characterization of local breeds to preserve their genomic variability.

**Electronic supplementary material:**

The online version of this article (doi:10.1186/1471-2164-15-601) contains supplementary material, which is available to authorized users.

## Background

The use of a relatively small number of international high-output or commercial breeds largely explains the increase in livestock productivity over the past decades. In parallel, the number of commercial populations is even decreasing due to consolidation of breeding stock and breeding companies [1]. While high productive breeds may not compete with low-input breeds in marginal regions or extensive production, FAO has expressed concern due to the shift from local breeds to high-output animals [2]. Local breeds may be more resistant than high-performance breeds to local diseases, may be better adapted to local climate, and may be adapted to poorer food quality [2, 3]. These characteristics of local breeds are very relevant for humans living in developing countries where local domestic animals are an important source of protein. Local breeds are also appreciated in developed countries for their cultural heritage value, and as producers of traditional and high quality meat products [4, 5]. Increasingly, local heritage breeds are recognized for their potential in sustainable or organic food production systems. Moreover, they represent a yardstick against which to compare highly selected breeds and allowing the detection of genes under selection [6]. Lastly, local breeds are claimed to harbour a large amount of the variation within livestock species [7, 8], and as such are recognized as important genetic reservoirs that need to be protected for future food security [9].

Despite all those inherent properties of local breeds, the long term survival of many of them is not assured [9]. Inbreeding is particularly relevant in local breeds that have low population numbers [5, 8]. The loss of genetic diversity within a breed due to drift and inbreeding can have direct consequences for reduction of survival, reproduction efficiency and capacity of adaptation to environmental changes [10]. The reduction in reproduction and growth rates is particularly relevant for local livestock breeds as it can directly lead to economic loss. Minimising inbreeding is, therefore, a major goal to guarantee the sustainability and maintenance of domestic populations of livestock species.

Genetic characterization of livestock breeds by applying genetic marker technology is needed to enhance breeding and to better direct biodiversity conservation strategies. In pigs, the Porcine SNP60 Bead-array [11] is a commercially available marker system extensively used in genetic studies (e.g. [12, 13]). More recently, whole-genome re-sequencing has emerged as an economically feasible tool for assessing genomic variation among populations [14]. In contrast to the commercially available SNP chip, the study of the whole genome sequence provides the opportunity of performing unbiased and comprehensive studies to characterize genetic diversity [15], regions of homozygosity [16], and scanning the pig genome to detect signatures of selection [17, 18]. The study of entire genomes increases the availability of information on neutral loci, and thereby the accuracy of estimates of demographically important parameters, such as the inbreeding coefficient (F) [19, 20]. Next generation sequencing (NGS) also allows for direct assessment of polymorphisms in coding regions that could have consequences in selective processes. For instance, genes involved in local adaptation, or alleles responsible for inbreeding depression can be analysed [19].

In this study, we first assess and compare genetic diversity of low-input breeds from Europe by integrating high-density SNP and re-sequencing data. Secondly, we explore the role of local breeds as reservoirs for genetic variation in a domesticated species. Finally, we assessed differences between local and commercial populations in terms of functional variation and explore evidences for inbreeding in local breeds that could lead to inbreeding depression.

## Results

We genotyped 12 local breeds from United Kingdom, Spain, Italy and Hungary (Table 1) with the Porcine SNP60 BeadChip [11]. SNP markers with more than 5% missing genotypes were excluded from the analysis. A total of 48,641 SNPs that could be mapped to autosomes on *Sus scrofa* build 10.2 [14] were finally used for the genetic diversity analysis. In addition, one or two representative genotyped pigs of these breeds, were re-sequenced to approximately 10x depth of coverage. The number of genomic variants, SNPs, and insertions or deletions (INDELs), varied greatly among the animals studied, ranging from 3.10 million in one Large White pig to 5.77 million in one British Saddleback pig. The number of variants and variability within exonic, intergenic, and intronic regions in all the re-sequenced animals is shown in the Additional file 1. In addition, a re-sequenced African Warthog was used as an out-group to deduce ancestral or derived status of alleles. Lastly, to characterize the distribution of alleles in non-western domestic populations, we made comparisons with a panel consisting of European and Asian Wild Boar and Chinese pigs.

**Table 1:** **Sampling information and analysis performed in each pig population**

Breed	Code	Category	Country	N	SNP	NGS
**British Saddleback**	BS	Local	UK	29	29	2
**Gloucester old spots**	GO	Local	UK	33	33	2
**Large black**	LB	Local	UK	30	30	1
**Middle white**	MW	Local	UK	27	27	2
**Tamworth**	TA	Local	UK	30	30	2
**Chato Murciano**	CM	Local	Spain	46	46	2
**Iberian pig**	IB	Local	Spain	29	29	2
**Cinta Senese**	CS	Local	Italy	13	13	1
**Casertana**	CT	Local	Italy	15	15	2
**Nera Siciliana**	NS	Local	Italy	15	15	0
**Calabrese**	CA	Local	Italy	15	15	1
**Mangalica**	MA	Local	Hungary	25	0	2
**Duroc**	DU	Commercial	International	2	0	2
**Large white**	LW	Commercial	International	2	0	2
**Landrace**	LR	Commercial	International	2	0	2
**Pietrain**	PI	Commercial	International	2	0	2
**Warthog**	-	Wild	-	2	0	2
**Wild boar**	WB	Wild	China	3	0	3
**Wild boar**	WB	Wild	The Netherlands	2	0	2

### Genetic diversity

**Figure 1:**
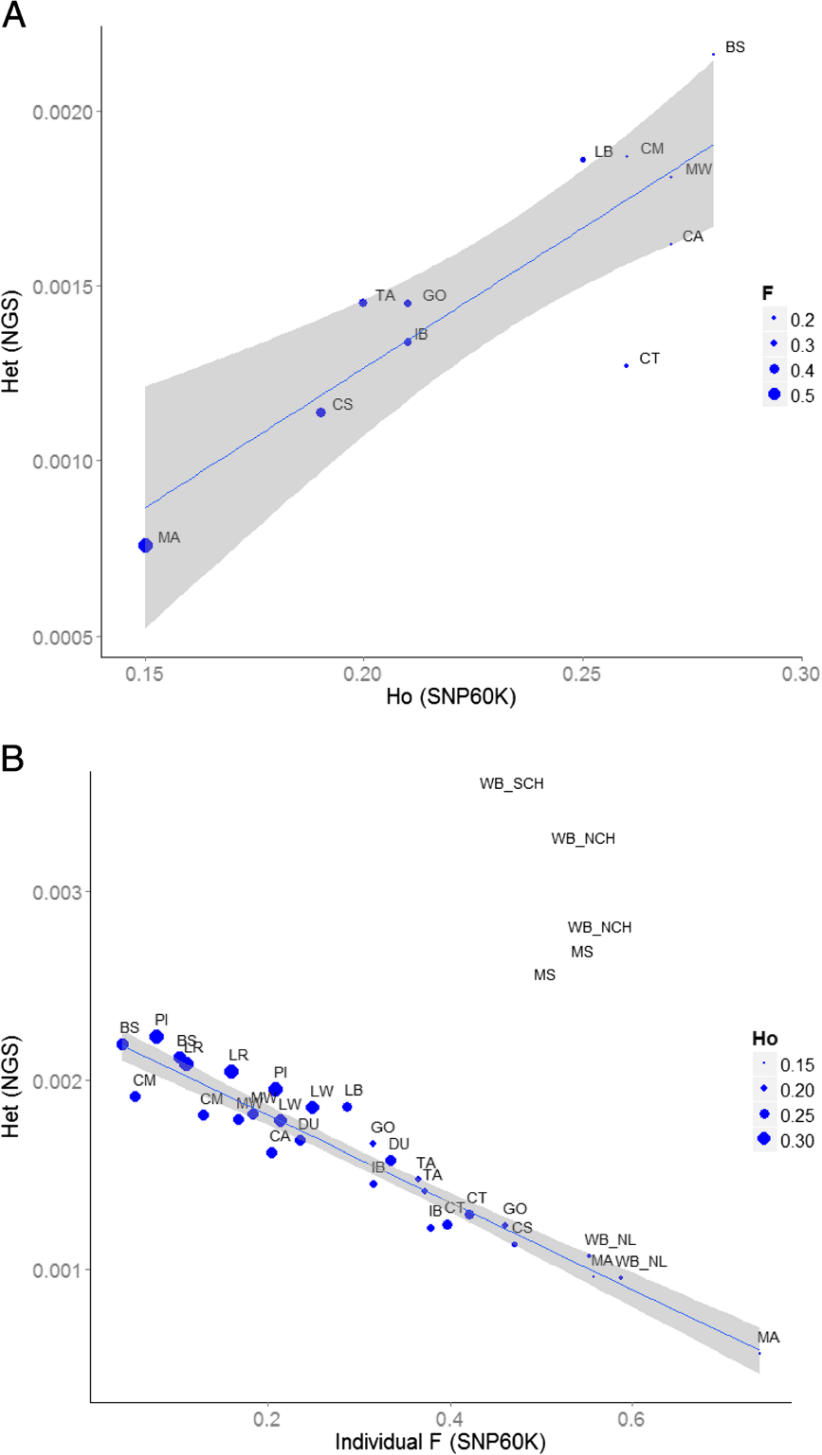
**Comparison of genetic diversity estimated with NGS and 60K-SNP data. (A)** Heterozygosity (Het) with NGS Vs. Observed heterozygosity (Ho) using 60K data at population level in local breeds. Each dot represents the average value in the populations. The size of the dots are proportional to the inbreeding coefficient (F) observed in the population. **(B)** Heterozygosity (Het) with NGS Vs. Inbreeding coefficient (F) using 60K data at individual level. Each dot represents a single pig. The size of the dots is proportional to the Ho using 60K at population level. The line that best fit the estimates in European pigs is displayed. The lack of correlation observed in Asian pigs indicates high ascertainment bias.

To estimate genetic diversity of the populations with 60K data, we used the expected and observed heterozygosity (He_60K and Ho_60K) computed with Genepop [21]. We also estimated individual inbreeding coefficient averaged in each population (F_60K) (Table 2). In addition, NGS data was used to calculate heterozygosity (h_NGS) [15]. The estimation of h_NGS was performed for each pig separately, and, when data from two individuals were available, the average was used as the estimation of h_NGS in the breed. The comparison of genetic diversity derived from 60K and NGS is shown in Table 2 and Figure 1. The study of European local breeds indicated that Mangalica has the lowest genetic diversity (He_60K = 0.19; h_NGS = 7.58E-04) and British Saddleback the highest (He_60K = 0.29; h_NGS = 2.16E-03). The two marker systems also agreed in the low genomic variability of Cinta Senese breed (He_60K = 0.20, h_NGS = 1.14E-03), high variability in Chato Murciano and Middle White (He_60K = 0.28-0.27; h_NGS = 1.87E-03-1.81E-03 respectively) and intermediate levels for Calabrese (He_60K = 0.24; h_NGS = 1.62E-03). Minor disagreements between the genotyping methods were observed in Iberian breed, with a lower estimate of genetic diversity based on NGS than on 60K data. In the English breeds Tamworth and Gloucester Old Spots the genetic diversity was low according to the 60K data (He_60K ≤ 0.21) but intermediate based on the NGS data (h_NGS ~ 1.45E-03). We observed a proportionally higher diversity in Casertana breed when 60K data was used at population level (Figure 1A). However, such disagreement between NGS and 60K was not observed at individual level (Figure 1B). This is explained by the existence of five Casertana pigs with negative inbreeding coefficient (F) values (see Additional file 2) that were analysed with 60K but not with NGS data. These Casertana pigs may have been recently crossed with other pigs resulting in an increased, but misleading, diversity to the overall population estimates using 60K data.

**Table 2:** **Genetic diversity parameters using Porcine SNP60 Beadchip (SNP) and Next generation sequence data (NGS)**

Continent	Category	Population	SNP			NGS
			*Ho*	*He*	*F*	*Het*
**Europe**	Local	BS	0.28	0.29	0.13	2.16E-03
**Europe**	Local	CA	0.27	0.24	0.17	1.62E-03
**Europe**	Local	CM	0.26	0.28	0.17	1.87E-03
**Europe**	Local	CS	0.19	0.2	0.41	1.14E-03
**Europe**	Local	CT	0.26	0.27	0.21	1.27E-03
**Europe**	Local	GO	0.21	0.21	0.34	1.45E-03
**Europe**	Local	IB	0.21	0.23	0.33	1.34E-03
**Europe**	Local	LB	0.25	0.25	0.23	1.86E-03
**Europe**	Local	MA	0.15	0.19	0.55	7.58E-04
**Europe**	Local	MW	0.27	0.27	0.16	1.81E-03
**Europe**	Local	TA	0.2	0.2	0.38	1.45E-03
**Europe**	Commercial	DU	0.26	0.27	0.29	1.63E-03
**Europe**	Commercial	LR	0.31	0.32	0.16	2.07E-03
**Europe**	Commercial	LW	0.3	0.31	0.19	1.82E-03
**Europe**	Commercial	PI	0.31	0.3	0.16	2.09E-03
**Europe**	Wild	WB_NL	0.17	0.19	0.55	1.01E-03
**Asia**	Wild	WB_NCH	0.17	0.18	0.53	2.96E-03
**Asia**	Wild	WB_SCH	0.21	0.22	0.45	3.49E-03
**Asia**	Local	MS	0.17	0.17	0.53	2.54E-03

Ho: Observed heterozygosity; He: expected heterozygosity; F: inbreeding coefficient; Het: Heterozygosity estimated using NGS data [15].

The study of parameters at the individual level ─ F_60K and h_NGS ─ allows a direct comparison between genetic diversity using the two marker systems (Figure 1B). In order to further assess the ascertainment bias, commercial and Asian pigs were included since the former may suffer less bias whereas Asian pigs are expected to have high ascertainment bias [14]. Not unexpectedly then, the major disagreement between 60K and NGS data along all the populations was found in the Asian pigs whose genetic diversity was largely underestimated by the 60K data (Figure 1B; Table 2). Apart from Asian pigs, we observed that English and commercial pigs tended to have higher genetic diversity in the estimates based on NGS than in 60K relative to the fitted line (Figure 1B). In contrast, pigs from Italy, Hungary and Spain showed lower than estimated genetic diversity based on NGS relative to the 60K SNP data. Despite these systematic deviation of the fitted model, the Pearson’s correlation coefficient computed using European pigs (both local and commercial pigs) was high and significant between Ho_60K and h_NGS (0.89, P < 0.01), and between He_60K and h_NGS (0.84, P < 0.01) at population level. A very high correlation between h_NGS and F_60K was observed when local pigs were analysed at individual level (-0.96, P < 0.01). The inclusion of the five Asian pigs in the analysis resulted in non-significant correlations lower than 0.2.

**Figure 2:**
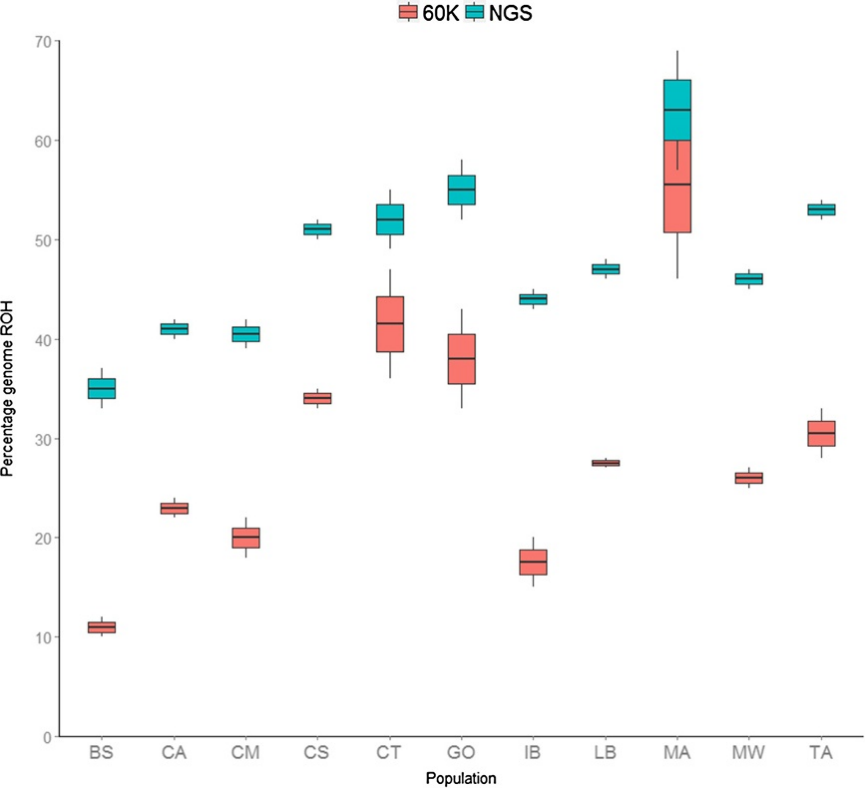
**Comparative analysis of the percentage of the genome covered by ROH in each breed.** Estimations using NGS are represented in blue and estimations using 60K data in red.

The number of Runs of Homozygosity (ROH) as well as their length varied greatly among populations as estimated from both 60K and NGS. In agreement with the genetic diversity estimates, all the analyses showed that the Mangalica breed had the highest proportion of the genome covered by ROH (Figure 2). The Italian breeds Casertana and Cinta Senese and the English breeds Tamworth and Gloucester Old Spots also had a high coverage of ROH (50-55% using NGS data). At the other end of the spectrum, the breed British Saddleback showed the lowest proportion (35%) followed by Calabrese and Chato Murciano (~40%). A high correlation between estimates of ROH was observed between estimates derived from NGS and 60K SNP data, although the 60K SNP data consistently underestimated the proportion of the genome covered by ROH (Figure 2). The comparison between the number and length of ROH using 60K and NGS revealed that 60K data tended to not discover short ROH and to overestimate the length of long ROH (Additional file 3). The correlation between length of ROH, estimated with NGS data, and the genetic diversity estimates F_60K and h_NGS was 0.79 and 0.84 respectively. The comparison of F value against the total length of ROH in the populations Calabrese, Chato Murciano, Casertana and Middle White encompassed pigs with a pattern of negative F values as well as shorter and lower number of ROH (Additional file 2).

### Functional significance of non-synonymous variants

Of all the SNPs discovered by NGS, an average of 0.17% was annotated as non-synonymous variants (Additional file 1). Considering all individuals, we observed a total of 16,409 different non-synonymous SNPs. All non-synonymous SNPs were analysed with Polyphen2 [22], that classifies mutations as benign and possible/probably damaging. In agreement with the genetic diversity estimations a high number of potentially damaging mutations is fixed in the breeds Mangalica, Cinta Senese, Tamworth and Gloucester Old Spots (Additional file 4). A phylogenetic tree of local breeds based on 16,409 non-synonymous SNPs resulted to be highly similar to the tree computed with 60K SNP data (Additional file 5). All English breeds clustered together and differentiated of the other European populations, which may reflect similarities in their demographic history. Calabrese and Chato Murciano breeds occupied an intermediate position between no introgressed European pigs and English breeds as a result of indirect Asian introgression from English and/or commercial pigs.

In order to find SNPs that potentially explain phenotypic differences between local populations and high-output pigs, we extracted all possible non-synonymous SNPs and we computed F_st_. Eight pigs derived from commercial elite lines (Duroc, Large White, Landrace and Pietrain) were considered as one population and each local breed was used separately to determine F_st_. We focussed on those non-synonymous SNPs that were fixed in commercial breeds and also in any local breed but with different allele, i.e. Fst = 1. Moreover, we explored the occurrence of ROH and published QTL overlapping these SNPs.

**Figure 3:**
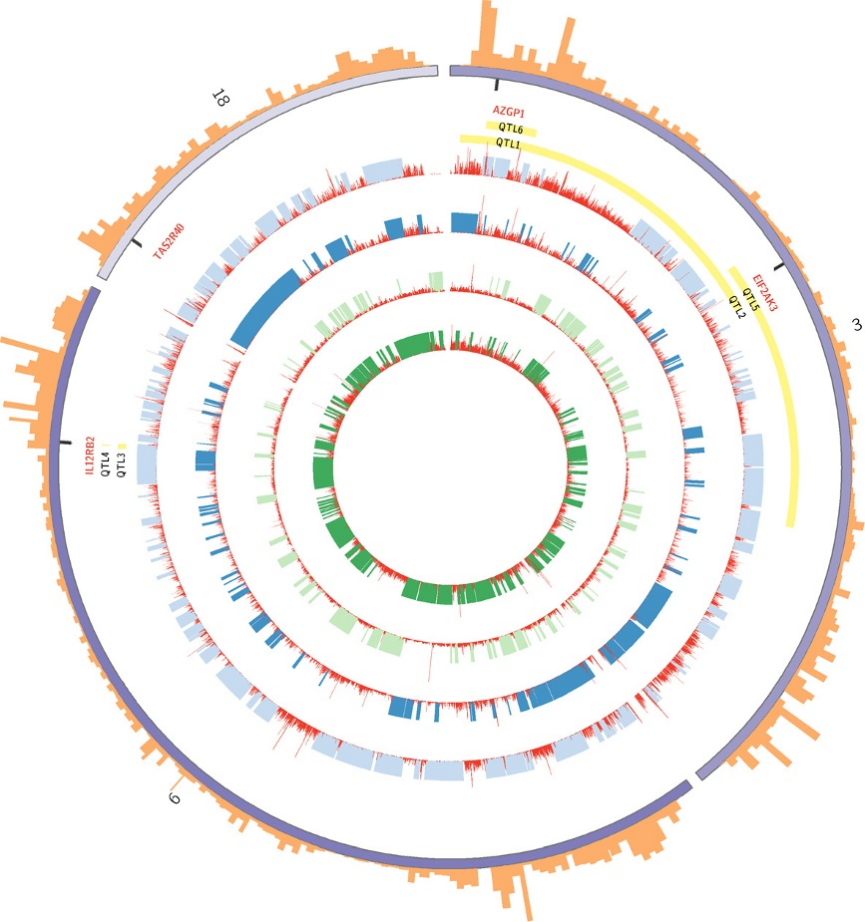
**Chromosomes 3, 6 and 18 are arranged circularly end-to-end using Circos**[23]**.** From inside to outside, the four inner rings display ROH (green and blue bars) and genetic diversity (red histograms) in Large White, Landrace, Mangalica and Tamworth respectively. Some QTLs overlapping any of the four genes studied are represented in yellow (QTL1: Abdominal fat weight; QTL2: Osteochondrosis score; QTL3: Intramuscular fat content; QTL4: Backfat thickness; QTL5: Feet and leg conformation; QTL6: Vertebra number. The outer ring represents the averaged high-density recombination map described by Tortereau et al. [24]*.*

This analysis revealed 99 non-synonymous SNPs with different fixed alleles in commercial and at least one of the local breeds, affecting 65 genes (Additional file 6). The comparison with a Warthog pig revealed that in 64% of fixed alleles it was the derived allele that was fixed in local pigs and 36% in commercial pigs. Among these 65 genes, we focused on those (i) with the two alleles –the ancestral and the derived– present in wild populations, (iii) those that were affected by several fixed SNPs and (iv) with a mutation classified by Polyphen2 (Additional file 6; Figure 3).

We observed a possible damaging mutation in the gene *AZGP1* in the breeds Mangalica, Cinta Senese and Gloucester Old Spot, as well as in European wild boar. This mutation overlaps with QTLs related with the number of vertebra, abdominal fat and ear morphology. It occupied a 50 kb genomic region where genetic diversity varied greatly among populations –from 0 to 5 times the averaged genetic diversity in the pig–. We observed two fixed SNPs within the gene *IL12RB2*, with Gloucester Old Spots, Middle White, Tamworth, Calabrese carrying the two ancestral alleles. Other local breeds such as British Saddleback and Chato Murciano were heterozygous at this locus, as were European and Asian wild pigs. This genomic region overlaps with meat and carcass quality QTLs such as back fat thickness and intramuscular fat content and the production QTLs for average daily gain and body weight. It also overlaps with ROH or low genetic diversity regions, except in British Saddleback and Large Black. A mutation classified as benign was observed within the gene *STAB1.* This gene codes for a protein involved in defence against bacterial infection by binding to bacteria and inducing phagocytic activity [25–27]. The allele was present in English breeds, Casertana and Asian pigs. *STAB1* overlaps with four QTLs related with CD4 and CD8 leukocyte percentage and ratio. The genetic diversity in this region is low, especially in commercial breeds with seven out of eight commercial breeds overlapping with a ROH. The two animals of the breed Mangalica, Chato Murciano and several English pigs were all homozygous for three derived alleles within the gene *EIF2AK3* while wild pigs had only one. The protein coded by this gene is involved in skeletal system development. The gene overlaps with QTL for feet and leg conformation and Osteochondrosis score. Local pigs carrying the derived allele have a ROH or a low genetic diversity in the 50 kb region overlapping this gene.

It must be considered that the large size of some QTLs and ROHs could lead into random associations with the SNPs under study. Therefore, we tested whether the patterns of overlapping between non-synonymous SNPs and QTLs were significantly different from random by a permutation test using 1,000 resamples (Additional file 7). The analysis showed a non-random overlapping between non-synonymous SNPs and exterior QTLs for ear weigh, area and size (P ≤ 0.002) and for leg conformation (P ≤ 0.007) as well as for average daily gain and body weight (P ≤ 0.01) which are categorized as production QTLs. The QTLs related to leukocyte number were also significantly overrepresented in the analysis (P ≤ 0.008). On the other hand, we are not able to discard a random association between SNPs and QTLs within the category meat and carcass quality and vertebra number (P > 0.05).

## Discussion

The advances in sequencing technologies now allows sequencing whole genomes in multiple individuals [19, 28]. However, the cost of this technology is still high, and budgets for conservation genetics research are limited. While high-density SNP panels allow the study of a representative sample size of a population at a much lower cost, there is a concern regarding the ascertainment bias implicit in the use of SNP chips [29]. This concern is even higher for local pig populations since they were not considered in the design the Porcine SNP60 Beadchip [11]. In this study, we found a high correlation between diversity estimates derived from the Illumina porcine 60SNP Beadchip and NGS data when local European breeds were analyzed. These results indicate that the Illumina porcine 60SNP Beadchip provides reliable estimates of genomic diversity for comparative studies between European populations, despite the expected bias. Nevertheless, English breeds showed greater diversity with NGS compared to 60K data than expected compared to expected values derived from all populations combined. These results may highlight the influence of historical breeding practices, whereby Asian pigs were used to improve local English pigs during the late 18^th^ and 19^th^ century [14, 30]. Despite the additional diversity found in English pigs owing to Asian introgression, some English pigs display high levels of ROH and potentially damaging mutations as a the result of recent inbreeding and could indicate that these breeds are prone to inbreeding depression.

SNP variants were annotated and potential deleterious effects were predicted with Polyphen2. Recessive deleterious alleles can be a major cause of inbreeding depression in populations with low genetic diversity [31]. In our study we find the largest number of putative deleterious mutations in those animals that also have the highest percentage of the genome covered by ROH and the lowest genetic diversity, i.e. Mangalica and Cinta Senese breeds, and in the breeds Tamworth and Gloucester Old Spots. Genomic diversity in these breeds was lower than almost all domestic and wild populations from Europe and Asia [16] corroborating the hypothesis that damaging mutations can accumulate, due to drift, in populations with high levels of inbreeding. A similar relation between genetic diversity and proportion of deleterious alleles has been described in human populations [32] and is thought to be caused by a less effective purifying selection as effective size decreases. This finding points out the need to develop conservation programs for endangered livestock populations that are very prone to high levels of inbreeding.

We found non-synonymous, high allele frequency differences (fixed for different alleles) at non-synonymous sites to be overrepresented in genes involved in immune response, anatomical development, behaviour, and sensory perception between commercial and local populations. Local breeds tend to be reared in traditional systems without being subjected to intense artificial selection (e.g. BLUP, GBLUP selection) as applied to commercial pig populations. As a result of years of different selection pressures and environments, genomic variations underlying phenotypic differences can be expected. We have specifically focussed on non-synonymous variants because they will alter the amino acid sequence of gene products, which may result in different phenotypes [33]. Although phenotypic change is expected to a large extent to result from regulation of genes, rather than differences in amino acid sequences, regulatory important variations are currently difficult to predict reliably and were therefore not considered in this study.

The gene AZGP1 stimulates lipid degradation in adipocytes and subsequently is considered a lipid-mobilizing factor [34]. This gene is linked with obesity in humans and its expression is inversely associated with body weight and percentage of body fat in mice and humans [35, 36]. In pigs, a 20 Mb QTL in chromosome 3 [37] for abdominal fat weight overlaps this gene. Mangalica, Cinta Senese and one European wild boar are homozygous for a derived allele annotated as probably damaging. This allele is absent in commercial pigs and also in some local pigs. The inferred status of the allele as ‘probably damaging’ may, for pig, rather result in having a large effect on the phenotype. Whereas pigs used to be bred for high fat deposition, in modern pig production systems lean meat is desired. AZGP1 also overlaps with a 16 Mb QTL for ear size, area and weight [38] and a 8.5 Mb QTL for vertebra number [39]. Ear morphology traits have been traditionally used to define breed standards. We observed a non-random overrepresentation of non-synonymous SNPs overlapping with QTLs related with ear morphology. This is in agreement with Wilkinson et al. [6] who found signatures of diversifying selection between pig breeds from Europe in genomic regions associated with ear morphology. Related to vertebra number, we found a fixed non-synonymous mutation in the Mangalica and heterozygous genotype in Iberian and Casertana breeds within the gene PLAG1 that has been related with stature in humans and cattle [40, 41]. Rubin et al. [18] concluded a strong signature of selection in the domestic pig genome at PLAG1. These data suggest that the mutations found in the genes AZGP1 and PLAG1 may represent signatures of different selection pressures between local breeds as Mangalica and commercial pigs. Another compelling example of potential differential selection between commercial and local populations is represented by the two mutations found in the bitter taste receptor TAS2R40. The high variability within the family of taste receptor genes has been suggested a consequence of adaptation of populations to specific dietary repertoires and environment [42], such as prevention of consumption of plant toxins [43].

It has been observed that selection for economically important traits tends to increase the susceptibility to environmental factors [44, 45]. In our study, ancestral mutations classified as benign in genes involved in immune related genes such as *IL12RB2* and *STAB1*, were observed in several local pigs. The IL12RB2 subunit plays an important role in Th1 cell differentiation that is critical for an effective immune response against different types of pathogens [46]. The three mutations observed in this gene overlap with important QTLs in pig production such as back fat thickness and intramuscular fat content [47, 48]. The fact that mutations in *IL12RB2* can lead to a defective IFN-gamma response to microorganisms [49, 50], suggests that disadvantageous genotypes could have been maintained in commercial populations.

The *EIF2AK3* gene overlaps with QTLs for osteochondrosis score [51] and feet and leg conformation [52]. Moreover, the permutation test using all the non-synonymous SNPs showed non-random overrepresentation of SNPs overlapping with QTLs for leg conformation. Interestingly, this gene encompasses functions of bone mineralization, chondrocyte development insulin secretion and fat cell differentiation and has being related with the Wolcott-Rallison syndrome in humans [53]. Leg weakness is a major concern in growing pigs raised under modern production systems and osteochondrosis is considered to be the primary cause of this syndrome. Indeed, forced selection for high growth capacity predisposes to these disorders due to an imbalance between the development of the skeletal system and muscle [54]. The allelic differences between local and commercial pigs within the *EIF2AK3* gene could underlie strong directional selection in commercial breeds. The fact that the same alleles are segregating in both wild boar and low-input breeds supports this hypothesis.

The genes discussed above had different fixed alleles for non-synonymous SNPs between commercial and local pigs. The presence of both alleles, the ancestral and the derived, in wild boars indicates that the variation was present before domestication. While differences in allele frequencies of SNPs in genes such as *AZGP1* and *TAS2R40* may underlie a rapid adaptation to different environments, it can also occur due to drift effects in small populations in the absence of selection, or even if the allele is in fact disadvantageous. The fixed alleles in *EIF2AK3* and *IL12RB2* could potentially result in disadvantageous phenotypes in high-output breeds owing to the strong artificial selection for production traits. We demonstrated that genetic variability found in wild populations is also being preserved in local breeds at genomic sites with potential phenotypic effect. This further highlights the importance of preserving local breeds as a source of genomic diversity that could be used in future selection programs of commercial pigs. However, the results presented also highlight high levels of ROHs, inbreeding and potentially damaging mutations that threat the future of local pig breeds, emphasizing the need of implementing conservation programmes to preserve the genomic variability of low-input breeds.

## Conclusions

In this study, we assessed genetic diversity of low-input breeds from different European regions by integrating high-density SNP and re-sequencing data. The comparison of the two marker system estimations provided insights for strategies to the genetic characterization of local breeds. Furthermore, the re-sequenced local pigs were compared with re-sequenced commercial pigs to report candidate mutations responsible for phenotypic divergence among those groups of breeds. We observed that local pig breeds are an important source of genomic variation within-species, and thereby, they represent a genomic stock that could be important for future adaptation to long-term changes in the environment or consumers preferences. However, high levels of inbreeding threaten the long term survival of some of the local breeds studied.

## Methods

### Animals and sampling and SNP genotyping

Blood samples from 315 unrelated domestic pigs were collected and DNA was extracted by using the QIAamp DNA blood spin kit (Qiagen Sciences). The study included domestic pigs that belonged to 12 local breeds from England, Spain, Italy and Hungary. Samples were genotyped using the Illumina Porcine 60K iSelect Beadchip [11] per manufacturers protocols. We included only SNPs mapped to one of the 18 autosomes on *Sus scrofa* build 10.2 and that had less than 5% missing genotypes. In addition, 1–2 animals of each local breed were selected for re-sequencing with the exception of the Nera Siciliana breed. We also re-sequenced eight individuals that belonged to the commercial, international pig breeds Duroc, Large White, Landrace and Pietrain. The samples used are detailed in Table 1.

### Ethics statement

DNA samples obtained from Chato Murciano pigs were obtained from blood samples collected by veterinarians. This procedure was approved by the Murcia University Ethics Committee and with the consent of the farmers. All the other samples were collected in the framework of the PigBioDiv1 and PigBioDiv2 projects. These DNA samples were obtained from blood samples collected by veterinarians according to national legislation, from tissue samples from animals obtained from the slaughterhouse or, in the case of wild boar, from animals culled within wildlife management programs.

### Sequencing alignment and SNP discovery

Library construction and re-sequencing of the samples was performed using 1–3 μg of genomic DNA following the Illumina library prepping protocols (Illumina Inc.). The library insert size ranged for 300–500 bp and fragments were sequenced from both sides yielding two times 100 bp mated sequences. Short read alignment was done against the *Sus scrofa* genome, build 10.2 [14] using Mosaik. The pigs were sequenced to a depth of approximately 10x. Further details on sequence mapping can be found in [16].

Archives in BAM format generated with the Mosaik Text function were used for the SNPs calling against the *Sus scrofa* genome, build 10.2. The mpileup function implemented in SAMtools v1.4-r985 [55] was used to obtain variant calls. Variations were filtered for a minimum genotype SNP and INDEL quality (20 and 50 respectively). Only variations based on a coverage in the range of 5x until twice the genome average were considered.

### Data analysis using high-density SNP genotyping

We used Genepop 4.2 [21] to compute the expected and observed heterozygosity. Inbreeding coefficient was calculated for all the individuals using PLINK 1.07 [56]. The ROHs were defined with PLINK 1.07 as regions of a minimum size of 10 kbp and encompassing 20 homozygous genomic sites, while allowing one heterozygous SNP. We predefined a minimum SNP density of 1 SNP/Mb and a largest possible gap between SNPs of 1 Mb to assure that the ROHs were not severely affected by the SNP density. Finally, we computed the Pearson’s correlation coefficient between length of ROHs and genetic diversity parameters in each breeds using R (http://www.r-project.org).

### Data analysis using NGS data

Heterozygosity was estimated for each individual as the number of heterozygous sites per 50 Kb-bin, corrected for total number of sites per bin [15]. Only bins that were sufficiently covered (per base at least a sequence depth of 7x and maximum of approximately 2 x average coverage) were considered. We obtained the heterozygosity for the population by averaging the individual heterozygosity of all individuals that belonged to that population. Correlations between 60K and NGS genomic diversity estimates were calculated using Pearson’s correlations in R environment. Graphics were obtained using the plotting system ggplot2 for R.

To estimate the ROH from re-sequencing data, we followed the procedure implemented by Bosse et el. [16], using a 100 kb sliding window. ROH were defined as a genomic region of at least 10 kb where the number of SNPs in an individual is less than expected based on the genomic average. Briefly, if the number of SNPs per bin = <0.25 x the genomic average, and if 10 or more consecutive bins showed a total SNP average lower than the total genomic average, they were extracted as candidates ROH.

ANNOVAR [57] was used to obtain the functional annotation (non-synonymous, synonymous, stop codon gain/loss, amino acid changes) of the genomic variants in each animal based on the pig reference genome (Swine Genome Sequencing Consortium Sscrofa10.2) obtained from the UCSC database (http://genome.ucsc.edu). For further analysis, only the non-synonymous sites were considered. The genes that overlap with the non-synonymous mutations were retrieved using Biomart [58].

The F_st_ value for all non-synonymous mutations was calculated using Genepop 4.2 [21]. For this analysis all the commercial pigs were considered as a single population while each local breed was considered separately. To reduce the number of SNPs to those that most likely represent the genetic basis of the phenotypic differences between commercial and local breeds, we only included in the study SNPs with F_st_ = 1 between the groups (i.e. fixed differences). Moreover, in order to avoid false positives, we exclusively considered those mutations that were homozygous in at least the two animals of the local breed. In the case of the local breeds that had only one animal re-sequenced or when one of the two animals of the breed showed missing data, the SNP was not considered for the functional analysis regardless its F_st_ value. Those SNPs with missing data in more than three commercial pigs were equally excluded.

The sequence of a re-sequenced Warthog was used to ascertain the alleles as ancestral or derived. The genotypes for those SNPs were also obtained from re-sequenced data from two domestic Meishan pigs, one wild boar from South China and two from North China and two European wild boars. The sequencing alignment and SNP discovery of these samples was the same as previously detailed.

Finally, we used the Polymorphism Phenotyping (PolyPhen2) algorithm [22] to predict phenotypic consequences of the non-synonymous sites. PolyPhen2 predicts whether a SNP is ‘benign’ , ‘possibly damaging’ or ‘probably damaging’ on the basis of evolutionary conservation, structure and sequence information.

### Availability of supporting data

The data sets supporting the results of this article are included within the article (and its additional files).

## Electronic supplementary material

Additional file 1: **Number of genomic variants within exonic, intergenic, and intronic regions.** (XLSX 12 KB)

Additional file 2: **Inbreeding coefficient Vs. Length of ROH using 60K data.** Each dot represents an individual and the size of the dots are proportional to number of ROH carried by the pig. The black line highlight the F = 0.00 value. (JPEG 1011 KB)

Additional file 3: **Example of ROH estimated with 60K and NGS in chromosomes SSC1 and SSC13.** The two lines of the same color represent the same animal, with the clearer color representing 60K estimation and the darker NGS results. The lack of detection of short ROH using 60K as well as overestimation of the length of long ROH is observed. From out to inside the circle: MA (orange), CT (green), TA (red), BS (yellow). (PNG 142 KB)

Additional file 4: **Detail of the mutations classified by Polyphen2.** Fixed mutations in local breeds with available Polyphen2 classification. Summary of the total number of potentially damaging mutations, total number of benign mutations and percentage of damaging mutations in each breed. (XLSX 845 KB)

Additional file 5: **Dendograms of the tested breeds based on pairwise F**_**st**_ **values using 60K and NGS data.** Dendrogram based on F_st_ pairwise between local breeds using 60K data; Dendrogram based on F_st_ pairwise between using 16.409 non-synonymous sites. (PDF 134 KB)

Additional file 6: **SNPs in coding sequence with extreme differences in allele frequencies (F**_**st**_ **= 1) between commercial and local pig populations.** (XLSX 18 KB)

Additional file 7: **Permutation analysis to test if patterns of overlapping between non-synonymous SNPs and QTLs were significantly different from random.** (XLSX 21 KB)

## References

[1] Muir WM, Wong GK-S, Zhang Y, Wang J, Groenen MAM, Crooijmans RPMA, Megens H-J, Zhang H, Okimoto R, Vereijken A, Jungerius A, Albers GAA, Lawley CT, Delany ME, MacEachern S, Cheng HH (2008). Genome-wide assessment of worldwide chicken SNP genetic diversity indicates significant absence of rare alleles in commercial breeds. Proc Natl Acad Sci U S A.

[2] (2012). Intergovernmental Technical Working Group on Animal Genetic Resources for Food and Agriculture. Seventh Session.

[3] Ramsay K (2002). Marketing rare breeds in Sub-Saharan Africa. Incent Meas Sustain use Conserv agrobiodiversity Exp lessons from South Africa.

[4] García-González DL, Aparicio R, Aparicio-Ruiz R (2013). Volatile and amino Acid profiling of dry cured hams from different Swine breeds and processing methods. Molecules.

[5] Herrero-Medrano JM, Megens HJ, Crooijmans RP, Abellaneda JM, Ramis G (2013). Farm-by-farm analysis of microsatellite, mtDNA and SNP genotype data reveals inbreeding and crossbreeding as threats to the survival of a native Spanish pig breed. Anim Genet.

[6] Wilkinson S, Lu ZH, Megens H-J, Archibald AL, Haley C, Jackson IJ, Groenen MAM, Crooijmans RPMA, Ogden R, Wiener P (2013). Signatures of diversifying selection in European pig breeds. PLoS Genet.

[7] Tapio I, Värv S, Bennewitz J, Maleviciute J, Fimland E, Grislis Z, Meuwissen THE, Miceikiene I, Olsaker I, Viinalass H, Vilkki J, Kantanen J (2006). Prioritization for conservation of northern European cattle breeds based on analysis of microsatellite data. Conserv Biol.

[8] Ollivier L (2009). European pig genetic diversity: a minireview. Animal.

[9] (2007). Global Plan of Action for Animal Genetic Resources and the Interlaken Declaration.

[10] Frankham R, Ralls K (1998). Conservation biology: inbreeding leads to extinction. Nature.

[11] Ramos AM, Crooijmans RPMA, Affara NA, Amaral AJ, Archibald AL, Beever JE, Bendixen C, Churcher C, Clark R, Dehais P, Hansen MS, Hedegaard J, Hu Z-L, Kerstens HH, Law AS, Megens H-J, Milan D, Nonneman DJ, Rohrer GA, Rothschild MF, Smith TPL, Schnabel RD, Van Tassell CP, Taylor JF, Wiedmann RT, Schook LB, Groenen M (2009). Design of a high density SNP genotyping assay in the pig using SNPs identified and characterized by next generation sequencing technology. PLoS One.

[12] Sahana G, Kadlecová V, Hornshøj H, Nielsen B, Christensen OF (2013). A genome-wide association scan in pig identifies novel regions associated with feed efficiency trait. J Anim Sci.

[13] Manunza A, Zidi A, Yeghoyan S, Balteanu VA, Carsai TC, Scherbakov O, Ramírez O, Eghbalsaied S, Castelló A, Mercadé A, Amills M (2013). A high throughput genotyping approach reveals distinctive autosomal genetic signatures for European and Near Eastern Wild Boar. PLoS One.

[14] Groenen MAM, Archibald AL, Uenishi H, Tuggle CK, Takeuchi Y, Rothschild MF, Rogel-Gaillard C, Park C, Milan D, Megens H-J, Li S, Larkin DM, Kim H, Frantz LAF, Caccamo M, Ahn H, Aken BL, Anselmo A, Anthon C, Auvil L, Badaoui B, Beattie CW, Bendixen C, Berman D, Blecha F, Blomberg J, Bolund L, Bosse M, Botti S, Bujie Z (2012). Analyses of pig genomes provide insight into porcine demography and evolution. Nature.

[15] Esteve-Codina A, Kofler R, Himmelbauer H, Ferretti L, Vivancos AP, Groenen MAM, Folch JM, Rodríguez MC, Pérez-Enciso M (2011). Partial short-read sequencing of a highly inbred Iberian pig and genomics inference thereof. Heredity (Edinb).

[16] Bosse M, Megens H-J, Madsen O, Paudel Y, Frantz LAF, Schook LB, Crooijmans RPMA, Groenen MAM (2012). Regions of Homozygosity in the porcine genome: consequence of demography and the recombination landscape. PLoS Genet.

[17] Amaral AJ, Ferretti L, Megens H-J, Crooijmans RPMA, Nie H, Ramos-Onsins SE, Perez-Enciso M, Schook LB, Groenen MAM (2011). Genome-wide footprints of pig domestication and selection revealed through massive parallel sequencing of pooled DNA. PLoS One.

[18] Rubin C-JC-J, Megens H-JH-J, Barrio AM, Maqbool K, Sayyab S, Schwochow D, Wang C, Carlborg O, Jern P, Jorgensen CB, Archibald AL, Fredholm M, Groenen MAM, Andersson L, Martinez Barrio A, Carlborg Ö, Jørgensen CB (2012). Strong signatures of selection in the domestic pig genome. Proc Natl Acad Sci U S A.

[19] Allendorf FW, Hohenlohe PA, Luikart G (2010). Genomics and the future of conservation genetics. Nat Rev Genet.

[20] Schraiber JG, Shih S, Slatkin M (2012). Genomic tests of variation in inbreeding among individuals and among chromosomes. Genetics.

[21] Rousset F (2008). genepop’007: a complete re-implementation of the genepop software for Windows and Linux. Mol Ecol Resour.

[22] Adzhubei IA, Schmidt S, Peshkin L, Ramensky VE, Gerasimova A, Bork P, Kondrashov AS, Sunyaev SR (2010). A method and server for predicting damaging missense mutations. Nat Methods.

[23] Krzywinski M, Schein J, Birol I, Connors J, Gascoyne R, Horsman D, Jones SJ, Marra MA (2009). Circos: an information aesthetic for comparative genomics. Genome Res.

[24] Tortereau F, Servin B, Frantz L, Megens H-J, Milan D, Rohrer G, Wiedmann R, Beever J, Archibald AL, Schook LB, Groenen MA (2012). A high density recombination map of the pig reveals a correlation between sex-specific recombination and GC content. BMC Genomics.

[25] Adachi H, Tsujimoto M (2002). FEEL-1, a novel scavenger receptor with in vitro bacteria-binding and angiogenesis-modulating activities. J Biol Chem.

[26] Kzhyshkowska J, Gratchev A, Brundiers H, Mamidi S, Krusell L, Goerdt S (2005). Phosphatidylinositide 3-kinase activity is required for stabilin-1-mediated endosomal transport of acLDL. Immunobiology.

[27] Kzhyshkowska J (2010). Multifunctional receptor stabilin-1 in homeostasis and disease. ScientificWorldJournal.

[28] Lachance J, Vernot B, Elbers CC, Ferwerda B, Froment A, Bodo J-M, Lema G, Fu W, Nyambo TB, Rebbeck TR, Zhang K, Akey JM, Tishkoff SA (2012). Evolutionary history and adaptation from high-coverage whole-genome sequences of diverse African hunter-gatherers. Cell.

[29] Albrechtsen A, Nielsen FC, Nielsen R (2010). Ascertainment biases in SNP chips affect measures of population divergence. Mol Biol Evol.

[30] Giuffra E, Kijas JM, Amarger V, Carlborg O, Jeon JT, Andersson L (2000). The origin of the domestic pig: independent domestication and subsequent introgression. Genetics.

[31] Hagenblad J, Olsson M, Parker HG, Ostrander EA, Ellegren H (2009). Population genomics of the inbred Scandinavian wolf. Mol Ecol.

[32] Lohmueller KE, Indap AR, Schmidt S, Boyko AR, Hernandez RD, Hubisz MJ, Sninsky JJ, White TJ, Sunyaev SR, Nielsen R, Clark AG, Bustamante CD (2008). Proportionally more deleterious genetic variation in European than in African populations. Nature.

[33] Strachan T, Read A, Wiley-Liss (1999). An overview of mutation, polymorphism, and DNA repair. Hum Mol Genet.

[34] Bao Y, Bing C, Hunter L, Jenkins JR, Wabitsch M, Trayhurn P (2005). Zinc-alpha2-glycoprotein, a lipid mobilizing factor, is expressed and secreted by human (SGBS) adipocytes. FEBS Lett.

[35] Gong F-Y, Zhang S-J, Deng J-Y, Zhu H-J, Pan H, Li N-S, Shi Y-F (2009). Zinc-alpha2-glycoprotein is involved in regulation of body weight through inhibition of lipogenic enzymes in adipose tissue. Int J Obes (Lond).

[36] Mracek T, Ding Q, Tzanavari T, Kos K, Pinkney J, Wilding J, Trayhurn P, Bing C (2010). The adipokine zinc-alpha2-glycoprotein (ZAG) is downregulated with fat mass expansion in obesity. Clin Endocrinol (Oxf).

[37] Beeckmann P, Schroffel J, Moser G, Bartenschlager H, Reiner G, Geldermann H (2003). Linkage and QTL mapping for Sus scrofa chromosome 3. J Anim Breed Genet.

[38] Ma J, Qi W, Ren D, Duan Y, Qiao R, Guo Y, Yang Z, Li L, Milan D, Ren J, Huang L (2009). A genome scan for quantitative trait loci affecting three ear traits in a White Duroc x Chinese Erhualian resource population. Anim Genet.

[39] Harmegnies N, Davin F, De Smet S, Buys N, Georges M, Coppieters W (2006). Results of a whole-genome quantitative trait locus scan for growth, carcass composition and meat quality in a porcine four-way cross. Anim Genet.

[40] Gudbjartsson DF, Walters GB, Thorleifsson G, Stefansson H, Halldorsson BV, Zusmanovich P, Sulem P, Thorlacius S, Gylfason A, Steinberg S, Helgadottir A, Ingason A, Steinthorsdottir V, Olafsdottir EJ, Olafsdottir GH, Jonsson T, Borch-Johnsen K, Hansen T, Andersen G, Jorgensen T, Pedersen O, Aben KK, Witjes JA, Swinkels DW, den Heijer M, Franke B, Verbeek ALM, Becker DM, Yanek LR, Becker LC (2008). Many sequence variants affecting diversity of adult human height. Nat Genet.

[41] Karim L, Takeda H, Lin L, Druet T, Arias JAC, Baurain D, Cambisano N, Davis SR, Farnir F, Grisart B, Harris BL, Keehan MD, Littlejohn MD, Spelman RJ, Georges M, Coppieters W (2011). Variants modulating the expression of a chromosome domain encompassing PLAG1 influence bovine stature. Nat Genet.

[42] Hayakawa T, Sugawara T, Go Y, Udono T, Hirai H, Imai H (2012). Eco-geographical diversification of bitter taste receptor genes (TAS2Rs) among subspecies of chimpanzees (Pan troglodytes). PLoS One.

[43] Glendinning JI (1994). Is the bitter rejection response always adaptive?. Physiol Behav.

[44] Van der Waaij EH (2004). A resource allocation model describing consequences of artificial selection under metabolic stress. J Anim Sci.

[45] Bloemhof S, Kause A, Knol EF, Van Arendonk JAM, Misztal I (2012). Heat stress effects on farrowing rate in sows: genetic parameter estimation using within-line and crossbred models. J Anim Sci.

[46] Koch MA, Thomas KR, Perdue NR, Smigiel KS, Srivastava S, Campbell DJ (2012). T-bet(+) Treg cells undergo abortive Th1 cell differentiation due to impaired expression of IL-12 receptor β2. Immunity.

[47] Ovilo C, Fernández A, Noguera JL, Barragán C, Letón R, Rodríguez C, Mercadé A, Alves E, Folch JM, Varona L, Toro M (2005). Fine mapping of porcine chromosome 6 QTL and LEPR effects on body composition in multiple generations of an Iberian by Landrace intercross. Genet Res.

[48] Muñoz G, Ovilo C, Silió L, Tomás A, Noguera JL, Rodríguez MC (2009). Single- and joint-population analyses of two experimental pig crosses to confirm quantitative trait loci on Sus scrofa chromosome 6 and leptin receptor effects on fatness and growth traits. J Anim Sci.

[49] Matsui E, Kaneko H, Fukao T, Teramoto T, Inoue R, Watanabe M, Kasahara K, Kondo N (1999). Mutations of the IL-12 receptor beta2 chain gene in atopic subjects. Biochem Biophys Res Commun.

[50] Poltorak A, Merlin T, Nielsen PJ, Sandra O, Smirnova I, Schupp I, Boehm T, Galanos C, Freudenberg MA (2001). A point mutation in the IL-12R beta 2 gene underlies the IL-12 unresponsiveness of Lps-defective C57BL/10ScCr mice. J Immunol.

[51] Laenoi W, Uddin MJ, Cinar MU, Grosse-Brinkhaus C, Tesfaye D, Jonas E, Scholz AM, Tholen E, Looft C, Wimmers K, Phatsara C, Juengst H, Sauerwein H, Mielenz M, Schellander K (2011). Quantitative trait loci analysis for leg weakness-related traits in a Duroc × Pietrain crossbred population. Genet Sel Evol.

[52] Uemoto Y, Sato S, Ohnishi C, Hirose K, Kameyama K, Fukawa K, Kudo O, Kobayashi E (2010). Quantitative trait loci for leg weakness traits in a Landrace purebred population. Anim Sci J.

[53] Mihci E, Türkkahraman D, Ellard S, Akçurin S, Bircan I (2012). Wolcott-Rallison syndrome due to a novel mutation (R491X) in EIF2AK3 gene. J Clin Res Pediatr Endocrinol.

[54] Arnbjerg J (2007). Effect of a low-growth rate on the frequency of osteochondrosis in Danish Landrace pigs (short communication). Arch Tierz, Dummerstorf.

[55] Li H, Handsaker B, Wysoker A, Fennell T, Ruan J, Homer N, Marth G, Abecasis G, Durbin R (2009). The sequence alignment/map format and SAMtools. Bioinformatics.

[56] Purcell S, Neale B, Todd-Brown K, Thomas L, Ferreira MAR, Bender D, Maller J, Sklar P, de Bakker PIW, Daly MJ, Sham PC (2007). PLINK: a tool set for whole-genome association and population-based linkage analyses. Am J Hum Genet.

[57] Wang K, Li M, Hakonarson H (2010). ANNOVAR: functional annotation of genetic variants from high-throughput sequencing data. Nucleic Acids Res.

[58] Haider S, Ballester B, Smedley D, Zhang J, Rice P, Kasprzyk A (2009). BioMart Central Portal--unified access to biological data. Nucleic Acids Res.

