# Supplementary figures and images for: Whole-genome sequence analysis reveals differences in population management and selection of European low-input pig breeds
Source: BMC Genomics. 2014 Jul 16;15(1):601. doi: 10.1186/1471-2164-15-601 (PMC4117957; doi:10.1186/1471-2164-15-601)

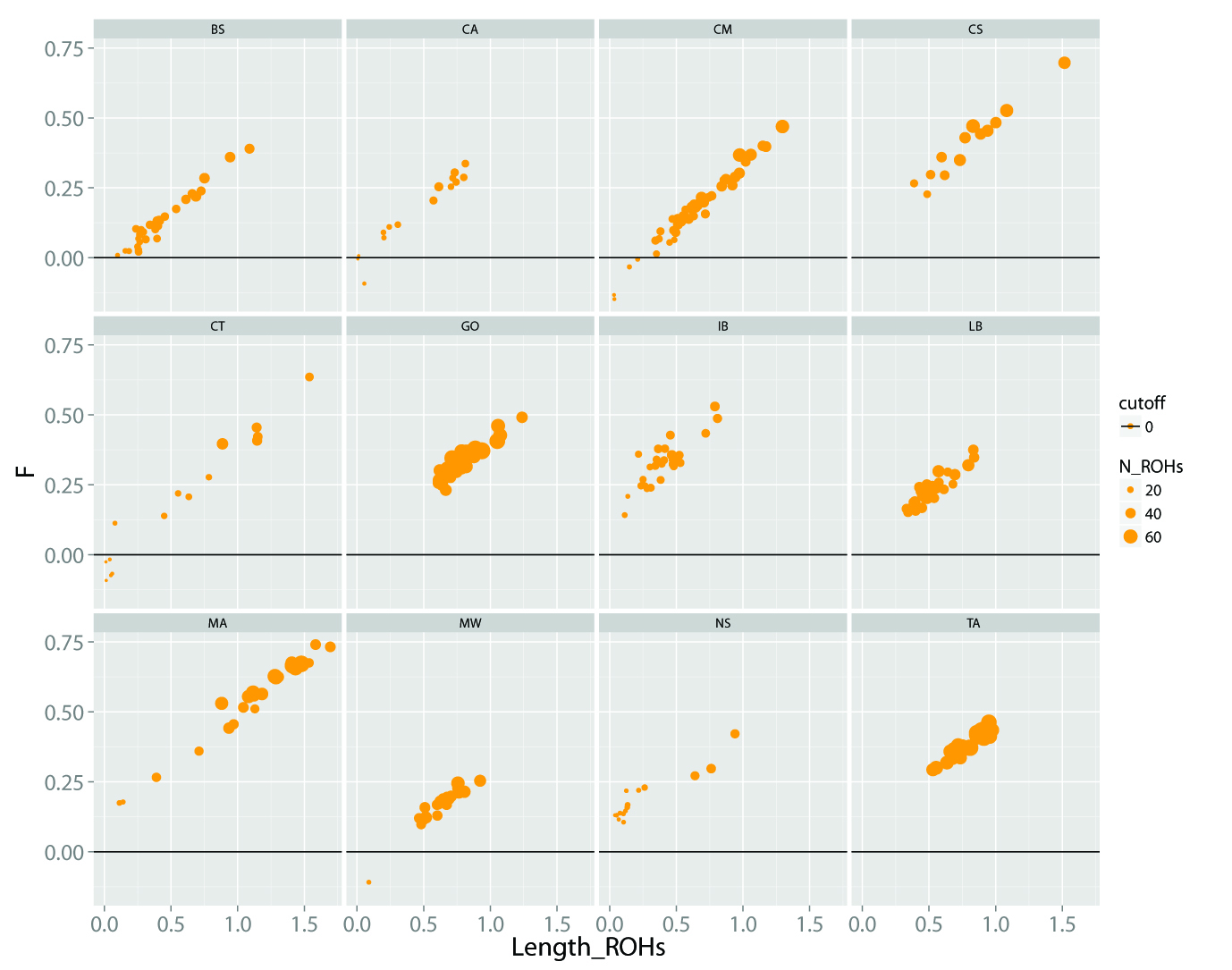

Supplement: Supplementary file 2 — Additional file 2: Inbreeding coefficient Vs. Length of ROH using 60K data. Each dot represents an individual and the size of the dots are proportional to number of ROH carried by the pig. The black line highlight the F = 0.00 value. (JPEG 1011 KB) [file 12864_2013_6301_MOESM2_ESM.jpeg]

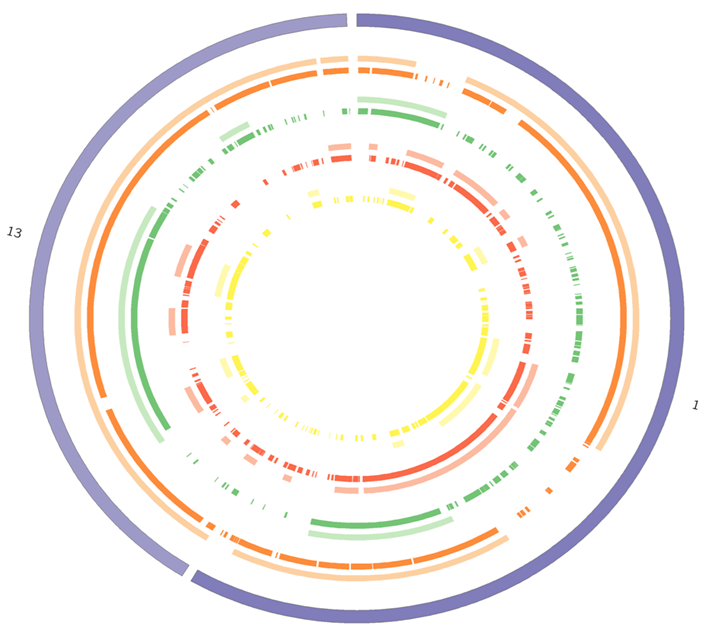

Supplement: Supplementary file 3 — Additional file 3: Example of ROH estimated with 60K and NGS in chromosomes SSC1 and SSC13. The two lines of the same color represent the same animal, with the clearer color representing 60K estimation and the darker NGS results. The lack of detection of short ROH using 60K as well as overestimation of the length of long ROH is observed. From out to inside the circle: MA (orange), CT (green), TA (red), BS (yellow). (PNG 142 KB) [file 12864_2013_6301_MOESM3_ESM.png]
