# Supplementary material for: Whole-genome sequence analysis reveals differences in population management and selection of European low-input pig breeds
Source: BMC Genomics. 2014 Jul 16;15(1):601. doi: 10.1186/1471-2164-15-601 (PMC4117957; doi:10.1186/1471-2164-15-601)
Supplement: Supplementary file 5 — Additional file 5: Dendograms of the tested breeds based on pairwise F st values using 60K and NGS data. Dendrogram based on Fst pairwise between local breeds using 60K data; Dendrogram based on Fst pairwise between using 16.409 non-synonymous sites. (PDF 134 KB) [file 12864_2013_6301_MOESM5_ESM.pdf]

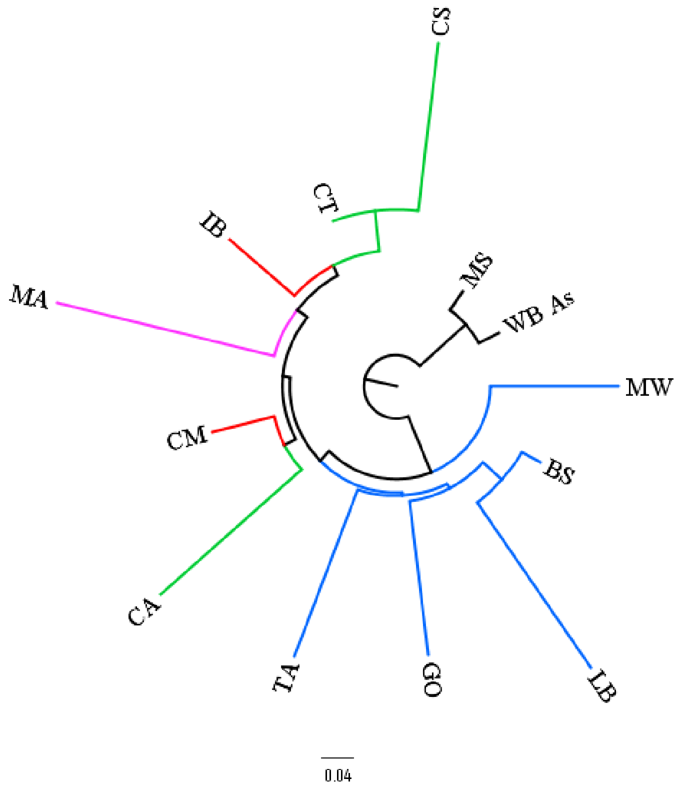

Dendrogram based on F_{st} pairwise between local breeds using 16.409 non-synonymous sites

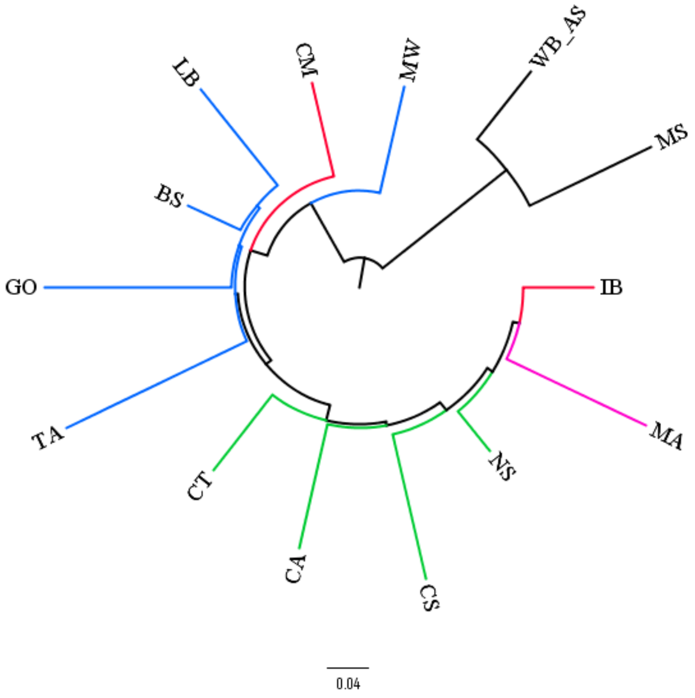

Dendrogram based on F_{st} pairwise between local breeds using 60K data
